# Clinical and demographic characteristics of patients presenting with post-infectious bronchial hyperresponsiveness at a pulmonology clinic

**DOI:** 10.3389/fmed.2025.1632712

**Published:** 2026-01-12

**Authors:** Nazlı Zeynep Uslu, Bensu Tanışman, Ebru Zeynep Delikanlı, Ayşenur Gençalp, Irem Karaman, Sebahat Dilek Torun, Merih Kalamanoğlu Balcı

**Affiliations:** 1 Department of Pulmonary Medicine, Bahcesehir University Faculty of Medicine, Istanbul, Türkiye; 2 Bahcesehir University School of Medicine, Istanbul, Türkiye; 3 Department of Public Health, Bahcesehir University Faculty of Medicine, Istanbul, Türkiye; 4 Department of Public Health, Yeditepe University Faculty of Medicine, Istanbul, Türkiye

**Keywords:** asthma, bronchial hyperresponsiveness, cough, respiratory tract infection, small airway disease

## Abstract

**Objective:**

This study aimed to characterize the clinical and demographic features of patients presenting with asthma-like symptoms following an upper respiratory tract infection (URI), despite no prior history of asthma. The goal of this study is to highlight this unrecognized clinical pattern and prevent misdiagnosis of asthma in this population.

**Methods:**

This cross-sectional study analyzed electronic medical records from 1,306 adult patients who presented to a pulmonology outpatient clinic with post-URI cough and associated symptoms. Extracted data included demographics, clinical manifestations, comorbidities, laboratory and spirometry results, and radiological imaging.

**Results:**

Prolonged cough was the presenting symptom in all patients, with the majority being female (62.9%) and having a mean age of 45.6 ± 12.3 years. The most common associated symptoms were sputum production (35.3%), post-nasal drip (34.6%), dyspnea (34.5%), and wheezing (34.2%). Demographically, older patients and ex-smokers had significantly longer symptom durations (≥3 weeks). Smokers had a significantly higher prevalence of wheezing and sputum production compared to non-smokers. Among patients with available spirometry (*n* = 594), 32.7% had reduced FEF_25-75_ values, suggesting small airway involvement. Older patients also showed a higher frequency of bronchovascular prominence on X-rays, which was significantly associated with dyspnea and elevated IgE levels.

**Conclusion:**

This study describes the characteristics of a specific patient population presenting with temporary post infectious bronchial hyperresponsiveness. Future studies should investigate the characteristics of these patients who are responsive to short-term treatment compared to those progressing to asthma. Recognizing this pattern may help prevent asthma misdiagnosis and optimize treatment strategies.

## Introduction

Upper respiratory tract infections (URIs) are ubiquitous (1), caused by various viral and bacterial pathogens leading to common illnesses such as acute bronchitis, the common cold, and influenza. While typically self-limiting, URIs induce airway inflammation, cough, and nasal congestion, with symptom duration averaging 7 to 10 days but sometimes persisting for up to 3 weeks. The inflammatory response associated with these infections leads to mucosal edema, vasodilation, and increased vascular permeability, contributing to airway irritation and prolonged symptomatology (2). Given the heterogeneity of URI presentations and outcomes, effective management often requires an interdisciplinary approach.

However, in a significant proportion of adults (estimated at 11 to 25%) following a respiratory infection, post-infectious respiratory symptoms persist beyond the typical recovery period, lasting longer than 3 weeks (3). This clinical pattern, including cough, sputum production, dyspnea, and wheezing, represents a substantial diagnostic challenge in pulmonology, particularly when occurring in individuals with no prior history of asthma. This widespread clinical dilemma is the ambiguity of differentiating transient post-infectious airway dysfunction from the emergence of new-onset or previously undiagnosed asthma (4).

The symptomatic overlap between transient Post-Infectious Bronchial Hyperresponsiveness (PIBHR) and asthma is a major factor driving high rates of diagnostic uncertainty. Recent population-based studies have revealed that up to one-third (33%) of adults who have received a physician diagnosis of asthma do not have active current asthma (5), with many cases likely being misclassified minor conditions or transient post-infectious syndromes that have remitted. The consequences of this misclassification create a critical pain point in healthcare delivery:

- **Overtreatment:** Patients with self-limiting PIBHR may be unnecessarily exposed to long-term inhaled corticosteroids (ICS) or bronchodilators. Studies show that a high percentage of misdiagnosed patients continue to take daily medication for a condition they do not have, incurring significant cost, side effects, and contributing to medical supply chain burden (6).
- **Undertreatment/Mismanagement:** Conversely, patients whose symptoms represent the true onset of chronic, unmasked asthma may not receive objective follow-up or adequate disease education if their condition is prematurely dismissed as “just a virus,” leading to lost opportunities to properly diagnose serious alternative conditions (5).

PIBHR refers to a transient increase in airway sensitivity and reactivity that develops following a resolved upper or lower respiratory tract infection, in individuals without prior chronic airway disease, and is characterized by reversible airflow limitation or cough-wheeze-dyspnea symptoms that resolve within weeks to months (4). Unlike asthma, PIBHR lacks persistent inflammation or variable airflow obstruction on serial testing; unlike post-viral cough, it involves demonstrable airway hyperresponsiveness rather than isolated cough; and unlike cough-variant asthma, it does not progress to chronic disease once the post-infectious inflammation subsides (4, 5).

The persistence of asthma-like symptoms following a URI in individuals without a prior asthma diagnosis may represent a distinct, self-limiting syndrome rather than undiagnosed asthma (7, 8). The temporal association between resolved infection and lingering respiratory symptoms suggests a post-infectious inflammatory process affecting airway function, rather than the onset of chronic disease.

The current knowledge gap is the lack of detailed, real-world data characterizing the specific clinical and demographic phenotype of patients presenting with PIBHR. This descriptive data is absent despite the high prevalence of the clinical dilemma. Such specific features could provide clinicians with tools to better differentiate the transient syndrome from evolving chronic disease at the initial presentation. Better characterization is essential for guiding initial management strategies (such as choosing between watchful waiting versus prompt objective testing), which contributes to precision medicine in this patient population.

Given these diagnostic complexities and the critical need for improved characterization to mitigate misdiagnosis, this study aims to:

1. Characterize the clinical and demographic features of patients presenting with asthma-like symptoms following a URI.
2. Identify objective clinical patterns, specifically small airway involvement and inflammatory biomarkers, that distinguish transient post-infectious symptoms from asthma-like profiles.
3. Provide descriptive data to assist clinicians in recognizing the phenotype of Post-Infectious Bronchial Hyperresponsiveness (PIBHR) to prevent asthma misdiagnosis and optimize treatment strategies.

## Methods

### Patients and study design

**Figure 1:**
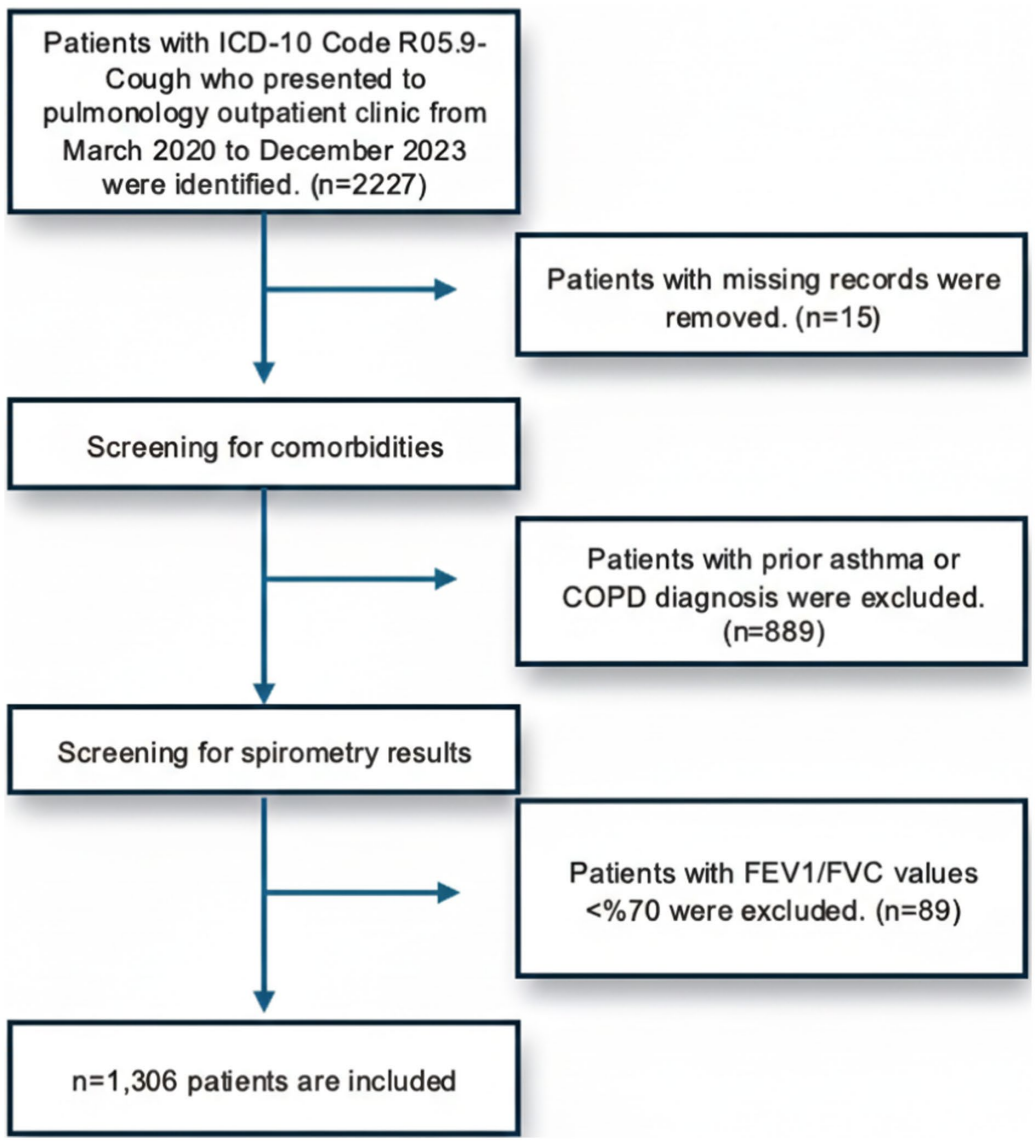
Flow diagram for the cross-sectional study of patients with post-URI symptoms.

This cross-sectional study included patients aged 18 to 85 years who visited the pulmonology outpatient clinic at a university-affiliated private hospital from March 2020 to December 2023 Cases with ICD-10 codes R05.9 (cough) identified and patients with a history of self-reported URI were selected. An asthma diagnosis was excluded in patients who did not meet Global Initiative for Asthma (GINA) criteria and exhibited a normal spirometry pattern (7). The study included those patients who had no prior asthma diagnosis and whose medical records were complete and provided sufficient data. A total of 1,306 adult patient files met these inclusion criteria and were included in the study. This study was approved by the Institutional Review Board (IRB) of Bahçeşehir University School of Medicine with the number of 2023-22/05. All data were anonymized to maintain patient confidentiality and privacy throughout the research process. Flow diagram of the study design has been shown in Figure 1.

### Inclusion criteria

Patients were included if they:

- Had the ICD code R05.9 (cough) presenting following self-reported URI at the time of visit.
- Reported preceding symptoms of URI (e.g., common cold, flu-like illness) but were not actively infected at the time of evaluation.
- Had persistent cough and at least one other asthma-like symptom (wheezing, dyspnea, sputum production, post-nasal drip).
- Did not meet asthma diagnostic criteria per GINA guidelines.
- In this retrospective setting, asthma was operationally excluded by: (i) no prior physician diagnosis of asthma; (ii) normal post-acute spirometry (FEV₁/FVC ≥ 0.70 with FEV₁ and FVC ≥ 80% predicted); and (iii) absence of documented bronchodilator reversibility when testing was available. Bronchial challenge testing, FeNO, and serial peak-flow variabilities were not routinely performed and therefore were unavailable for case adjudication.

To distinguish post-infectious symptoms from an ongoing URI, patients were only included if:

- Their symptoms persisted beyond the typical acute infection period (~7 days).
- They lacked signs of active infection:
- Neutrophil and lymphocyte counts within the reference range.
- No radiographic evidence of infection (consolidation, ground-glass opacities, reticulonodular patterns, lymphadenopathy, or pleural effusion) on chest X-rays.

### Exclusion criteria

- Patients with a prior asthma or COPD diagnosis.
- Patients with ongoing respiratory infections (based on clinical, X-ray, and blood findings).
- Patients with missing medical records.

### Data collection

Data was retrospectively collected by extraction from the hospital’s Electronic Medical Records (EMR). The extraction was performed using a standardized data collection form developed by the research team to ensure the consistency and accuracy of data recorded from the EMR. For each patient, sociodemographic information, including age, sex, and clinical background such as smoking status, history of allergy, family history of asthma, and any chronic diseases, was recorded.

Additional most common reported symptoms such as cough, wheezing, post-nasal drip, sputum production, and dyspnea, and the duration from symptom onset to the clinic visit with a three-week cut-off point (for distinguishing acute vs. subacute cough) were recorded (8). Physical examination findings of rales, rhonchi, and wheezing, along with chest X-ray results, which were classified into three categories according to radiologists’ reports at the time of imaging: normal findings, increased density, and prominent bronchovascular markings were recorded.

Blood parameters, including white blood cell count (WBC), hemoglobin (HGB), hematocrit level (HCT), mean corpuscular volume (MCV), immunoglobulin E (IgE), eosinophil count (EOS), neutrophil count (NEUT), lymphocyte count (LYM), and C-reactive protein level (CRP), were classified as “within the reference range,” “below the reference range,” or “above the reference range” based on the biochemical laboratory reference values of our hospital. The reference ranges were as follows: WBC (4.23–10.2 K/μL), HGB (12.2–16.2 g/dL), HCT (37.7–47.9%), MCV (80–100 fL), IgE (0–100 IU/mL), EOS (0.04–0.36 K/μL), NEUT (1.56–6.13 K/μL), LYM (1.18–3.57 K/μL) and CRP (0–8.2 mg/L).

Pathogen testing (e.g., PCR panels, cultures, serology) was not routinely performed as part of outpatient evaluation; therefore, the etiologic agent (viral vs. bacterial) could not be confirmed. Infection type was inferred from clinical presentation and physician notes, with inclusion restricted to patients who were clinically recovered from the acute infectious phase.

Comorbidities were categorized into cardiovascular diseases (e.g., hypertension, acute coronary syndrome, cardiomyopathy), rheumatic diseases [e.g., systemic lupus erythematosus (SLE), ankylosing spondylitis (AS), rheumatoid arthritis (RA)], gastroesophageal reflux disease (GERD), diabetes mellitus, and thyroid disorders (e.g., hypothyroidism, Hashimoto’s disease). These conditions were identified based on patient history and medical records to assess their potential association, as they may influence symptom presentation or persistence.

For lung function assessment, we identified patients who underwent pulmonary function tests (PFTs) at the time of admission, using the hospital’s electronic archive. Pulmonary function testing was conducted in line with the latest guidelines from the American Thoracic Society (ATS) and the European Respiratory Society (ERS). The severity of airway obstruction was classified, and its association with various clinical and demographic factors was analyzed. Forced expiratory volume in 1 second (FEV1), forced vital capacity (FVC), FEV1/FVC ratio, the average forced expiratory flow rate at 25–75% of the vital capacity (FEF25-75%) were recorded and interpreted according to latest ERS/ATS guidelines. Spirometry was conducted after the acute phase of the URI to prevent active infection from affecting the results. An FEV₁/FVC ratio below 0.70 was used to identify obstructive ventilatory defects, as recommended by the Global Initiative for Chronic Obstructive Lung Disease (GOLD) guidelines. Small Airways Disease (SAD) was defined using spirometric parameters, with a reduced FEF_25−_₇₅% (below 75%) and a preserved FEV₁/FVC ratio (above 0.70) indicating the presence of SAD (9).

### Statistical analysis

Data were initially recorded in a Microsoft Excel (Microsoft, 2018) spreadsheet and then imported into IBM SPSS Statistics version 25.0 (IBM Corp, 2017) for analysis (10, 11). Descriptive findings of the patients’ demographic and clinical characteristics were reported as mean and standard deviation, minimum and maximum values for numerical variables and as frequency distributions (number and percentages) for categorical variables. Associations between categorical demographic and clinical factors and persistent asthma-like symptoms were assessed using chi-square tests and Fisher exact test. Differences in means for continuous variables were assessed using the Student’s t test. A *p* value of less than 0.05 was considered as statistically significant at 95% confidence level.

## Results

### General characteristics of study population

This study included 1,306 patients, of whom 821 (62.9%) were women. The most prevalent age group was 35–44 years, comprising 382 individuals (29.2%), followed by the 25–34 age group with 372 individuals (28.5%), and the 45–54 age group with 229 individuals (17.5%) (Table 1).

**Table 1:** Demographics and clinical characteristics of the study population.

Characteristic	Value (*n*, %)
Total patients	1,306 (100%)
Age (mean ± SD)	45.6 ± 12.3 years
Age group: 18–24 years	80 (6.1%)
Age group: 25–34 years	372 (28.5%)
Age group: 35–44 years	382 (29.2%)
Age group: 45–54 years	229 (17.5%)
Age group: 55–64 years	143 (10.9%)
Age group: 65 + years	100 (7.6%)
Female (*n*, %)	821 (62.9%)
Smokers (*n*, %)	539 (41.3%)
Ex-Smokers (*n*, %)	83(6.4%)
Non-Smokers (*n*, %)	525 (40.2%)
Reported Allergies (*n*, %)	870 (66.6%)
History of gastroesophageal reflux disease (*n*, %)	49 (3.8%)
History of cardiovascular disease (*n*, %)	134 (10.3%)
History of rheumatic disease (*n*, %)	36 (2.8%)
History of diabetes mellitus (*n*, %)	56 (4.3%)
History of thyroid disease (*n*, %)	71 (5.4%)
Symptoms and findings	
Duration of symptoms < 3 weeks (*n*, %)	927(71.0%)
Duration of symptoms ≥ 3 weeks (*n*, %)	379(29.0%)
Sputum production (*n*, %)	461 (35.3%)
Post-nasal drip (*n*, %)	452 (34.6%)
Dyspnea (*n*, %)	450 (34.5%)
Wheezing (*n*, %)	446 (34.2%)
Small Airways Disease (SAD) (*n*, %)	200 (15.3)
Abnormal X-ray findings (*n*, %)	100 (7.7%)

Symptom duration was categorized as either less or longer than three weeks, given that subacute cough is defined as lasting longer than three weeks. There was a significant difference between age groups and symptom duration (*p* = 0.006), with younger individuals showing a higher proportion of symptoms lasting less than three weeks, while 40% of those aged 65 and above reported symptoms persisting beyond three weeks (Table 2). Male patients had a significantly higher proportion of symptoms lasting more than three weeks compared to females (*p* = 0.025) (Table 2). Symptom duration was significantly associated with sputum production (*p* = 0.005), where patients reporting sputum had a lower proportion of symptoms lasting three weeks or longer (24.3%) compared to those without sputum (31.6%). Conversely, those experiencing dyspnea showed a significantly higher proportion of symptoms lasting three weeks or longer (34.2%) compared to those without dyspnea (26.3%) (*p* = 0.003). Regarding smoking status, there was a significant association with longer symptom duration (*p* = 0.019). Ex-smokers were more likely to have symptoms persisting beyond three weeks compared to non-smokers and current smokers. Notably, no significant relationships were found between symptom duration and the presence of wheezing, nor between symptom duration and comorbid conditions such as cardiological or rheumatological diseases, diabetes mellitus, or thyroid diseases.

**Table 2:** Association between symptoms duration and demographics and clinical characteristics of the study population.

Clinical/Demographic characteristics	< 3 weeks (*n*, %)	≥ 3 weeks (*n*, %)	*p*-value
Gender			0.025*
Female	565 (68.8%)	256 (31.2%)	
Male	362 (74.6%)	123 (25.4%)	
Smoking status			0.019*
Non-smoker	372 (70.9%)	153 (29.1%)	
Smoker	393 (72.9%)	146 (27.1%)	
Former smoker	48 (57.8%)	35 (42.2%)	
Wheezing			0.646
Present	313 (70.2%)	133 (29.8%)	
Absent	614 (71.4%)	246 (28.6%)	
Post-nasal drip			0.152
Present	332 (73.5%)	120 (26.5%)	
Absent	595 (69.7%)	259 (30.3%)	
Sputum production			0.005**
Present	349 (75.7%)	112 (24.3%)	
Absent	578 (68.4%)	267 (31.6%)	
Dyspnea			0.003**
Present	296 (65.8%)	154 (34.2%)	
Absent	631 (73.7%)	225 (26.3%)	
Age	41.18 (SD: 13.558)	43.48 (SD: 14.026)	0.006**

*p ≤ 0.05, **p ≤ 0.01.

### Comorbid diseases

Patients with respiratory diseases such as asthma and COPD were excluded from the study. Comorbidities were categorized as cardiovascular diseases (e.g., hypertension, acute coronary syndrome, cardiomyopathy), rheumatic diseases (e.g., Systemic Lupus Erythematosus, Ankylosing Spondylitis, Rheumatoid Arthritis) Gastroesophageal Reflux Disease (GERD), diabetes mellitus, and thyroid diseases. Most individuals did not report these specific health conditions, with GERD and rheumatic diseases being the least common, at 3% (*n* = 49) and 2.8% (*n* = 36), respectively. Cardiovascular diseases (10.3% or *n* = 134) and hypothyroidism/Hashimoto’s disease (5.4% or *n* = 71) were relatively more prevalent in the study population. GERD was significantly associated with post-nasal drip (*p* < 0.001) but not with other symptoms.

### History of allergy

Eight hundred and seventy patients (66.6%), reported a history of allergy, with the highest allergy rates observed in females (69.3%) compared to males (62.1%) (*p* < 0.05) (Supplementary Table 2). Among those with allergies, 39.8% had post-nasal drip, and 37.2% reported sputum production, which were significantly more common than patients with no history of allergies (*p* < 0.05). Wheezing, shortness of breath, and other physical examination findings (rales, rhonchi, wheezing) did not differ significantly between those with and without allergies. Most individuals with allergies had normal X-ray findings (89.8%), with no significant association between allergies and the presence of SAD. Elevated IgE levels were observed in 41.4% of individuals with allergies.

### Lung function and spirometry results

Among 1,306 patients, spirometry results were available for 594 patients, and all of them had FEV1/FVC ratio of greater than 0.70 and both FEV1 and FVC above 80%, which have been interpreted as normal. Among the 594 patients 200 had reduced FEF25-75 value and identified with Small Airways Disease (9). Patients with this small airway involvement were significantly older (48.1 vs. 44.2 years, *p* = 0.006) and reported higher rates of dyspnea (41.5% vs. 31.0%, *p* = 0.012) and bronchovascular prominence on X-ray (*p* = 0.04) compared to those with normal spirometry (Table 3).

**Table 3:** Clinical features stratified by small airway involvement (represented with reduced FEF25-75).

Characteristic	Total with spirometry (*n* = 594)	Normal spirometry (*n* = 394)	Reduced FEF_25-75_ (*n* = 200)	*p*-value
Age (Mean ± SD)	45.6 ± 12.3	44.2 ± 11.9	48.1 ± 13.1	0.006**
Symptom duration ≥ 3 weeks	29.0%	27.2%	32.5%	0.18
Dyspnea (symptom)	34.5%	31.0%	41.5%	0.012*
Sputum production	35.3%	33.0%	39.5%	0.11
Smoking status				
Non-smoker	40.2%	42.1%	36.5%	0.19
Current/ex-smoker	59.8%	57.9%	63.5%	
Bronchovascular prominence (X-ray)	7.7%	6.1%	11.0%	0.04*

*p ≤ 0.05, **p ≤ 0.01, ***p ≤ 0.001.

### Chest X-ray findings and blood parameters

Chest X-ray reports were available for 857 patients. Among these, 757 (58%) were reported as normal. Abnormal findings were observed in 100 patients (7.7%), with the most common radiological feature being bronchovascular prominence, reported in 81 cases (6.2%). Additionally, 20 patients (1.5%) exhibited increased opacity, while cardiomegaly was identified in one patient (0.1%). There was no significant relationship between smoking status and X-ray results, nor was there an association between symptom duration and abnormal chest X-ray findings. However, a significant association was found between age groups and bronchovascular prominence on X-rays with older age groups (45–54, 55–64, 65+) showing higher rates (*p* < 0.001) (Supplementary Table 3). Dyspnea was also significantly associated with bronchovascular prominence (*p* = 0.002), and elevated IgE levels showed a significant association with increased bronchovascular prominence on X-rays (*p* = 0.035).

### Blood parameters

WBC levels were recorded for 726 patients, with 12 (1.7%) exceeding the reference range. Neutrophil counts were available for 725 patients, and lymphocyte counts for 726 patients, with all values falling within the reference range. Out of the 728 patients with recorded eosinophil data, 112 (15.4%) had elevated values (Supplementary Table 4). CRP levels were available for 666 patients, among whom 187 (28.1%) exhibited elevated levels.

IgE levels were measured in 243 patients, with 94 (38.7%) showing values above the reference range. No significant association was identified between IgE, eosinophil counts, or CRP levels and the duration of symptoms. Significant associations were observed between smoking status and the levels of Hgb, HCT, and IgE (Supplementary Table 4). Nonsmokers had a higher proportion of Hgb, HCT, and IgE levels within the reference range, while smokers showed higher percentages of Hgb, HCT, and IgE levels above the reference range. However, when comparing Hgb, HCT, eosinophils, and IgE levels with allergy history, no significant association was found. The duration of symptoms did not show any significant association with these blood parameters.

When stratified by inflammatory profiles, patients with asthma-associated biomarkers (elevated IgE or eosinophils) exhibited significantly higher frequencies of wheezing (*p* < 0.001) and bronchovascular prominence (*p* = 0.035) compared to those with normal biomarker levels, despite similar symptom durations (Table 4).

**Table 4:** Comparison of patients clinical characteristics with asthma-associated biomarkers (elevated IgE or eosinophils) vs. normal biomarkers.

Clinical characteristic	Normal biomarkers (normal IgE & eosinophils)	Asthma-associated biomarkers (elevated IgE or eosinophils)	*p*-value
Symptom duration ≥ 3 weeks	28.1%	29.8%	0.65
Wheezing (Symptom)	31.5%	42.1%	<0.001***
Bronchovascular prominence (X-ray)	6.8%	13.4%	0.035*
History of atopy/allergy	58.2%	76.5%	<0.001***
Sputum production	33.1%	38.2%	0.21

*p ≤ 0.05, **p ≤ 0.01, ***p ≤ 0.001.

## Discussion

Post-infectious asthma-like syndromes following URIs in individuals without a prior asthma diagnosis present as transient respiratory conditions with symptoms such as cough, wheezing, dyspnea, sputum production, and post-nasal drip, yet do not fulfill the diagnostic criteria for asthma. This study provides a detailed clinical and demographic phenotype of this patient cohort (Objective 1), which is essential for differentiation (Objective 2).

Our findings suggest that PIBHR presents as a distinct clinical phenotype characterized by small airway dysfunction rather than the large airway obstruction typical of established asthma. While all patients in our study had ‘normal’ FEV1/FVC ratios by standard definition, Table 3 reveals that 33.7% exhibited reduced FEF25-75 values. This reduction was significantly associated with older age and dyspnea, yet notably, it occurred independently of symptom duration. This supports the hypothesis that post-infectious inflammation may preferentially target the distal airways (‘the silent zone’) (12), creating a sensation of dyspnea and cough without satisfying the spirometric criteria for asthma (13, 14). Clinicians seeing this pattern—normal FEV1 but reduced FEF25-75—should consider PIBHR as a primary differential, particularly in non-atopic patients.

To address the challenge of differentiating this transient condition from undiagnosed asthma (Objective 2), we stratified patients based on inflammatory biomarkers. The fact that patients with an ‘asthma-compatible’ profile (elevated IgE or eosinophilia) presented with significantly higher rates of clinical wheezing and radiological bronchovascular prominence aligns with established evidence linking IgE-mediated inflammation to structural airway changes and hyperreactivity (15). This contrast is vital for raising awareness: the presence of objective wheezing combined with Type-2 inflammatory markers should prompt a rigorous investigation for asthma (7). Conversely, the absence of these features in a patient with post-URI cough suggests a self-limiting course, even if symptoms feel “asthma-like” to the patient.

These findings have immediate implications for clinical management and the prevention of misdiagnosis. The ‘PIBHR phenotype’ identified here (cough >3 weeks, reduced FEF25-75, normal IgE/Eosinophils) likely represents the population most at risk of overtreatment with long-term asthma controllers (16). Recognizing this pattern allows clinicians to confidently employ ‘watchful waiting’ or short-course therapy rather than committing patients to chronic disease management. Guidelines support the use of short-term inhaled corticosteroids (ICS) for subacute post-infectious cough (8), but therapeutic gain is modest (17, 18), and indiscriminate use should be avoided. Conversely, the specific subset of patients with prominent bronchovascular markings and elevated IgE represents a higher-risk group that may require earlier objective testing (e.g., bronchial challenge) to rule out underlying asthma.

### Addressing general management

Post-infectious cough, typically resolving within eight weeks, is linked to heightened cough reflex sensitivity and temporary bronchial hyperresponsiveness, often with eosinophilia but without other asthma features (19, 20). Viral infections can prolong cough duration, but most cases resolve within six weeks. Infection-induced bronchial hyperresponsiveness can similarly cause a subacute cough (3–8 weeks). Beta-2 sympathomimetics and inhaled corticosteroids may shorten cough duration, likely due to variable bronchial hyperresponsiveness (21, 22). If chronic cough persists without airway obstruction but responds to this therapy, cough-variant asthma should be considered, underscoring its overlap with post-infectious airway dysfunction (23).

In patients presenting with persistent symptoms following the resolution of an URI, it is important to consider that CRP levels may remain elevated beyond the acute phase. While moderately elevated CRP values (10–60 mg/L) are common during the initial days of a viral URI, peaking around days 2–4, these levels typically decline as the infection resolves (24). However, studies with asthma patients have shown that increased serum high-sensitivity CRP (hs-CRP) concentrations correlate positively with asthma severity, suggesting that CRP can serve as a marker for ongoing airway inflammation even after the acute infection has subsided (25). Therefore, in patients with lingering respiratory symptoms post-URI, persistently elevated CRP levels may indicate continued inflammatory processes, necessitating further evaluation and management (24, 25).

Smoking significantly influences the persistence of respiratory symptoms following upper respiratory tract infections (URIs), with both current and former smokers exhibiting prolonged symptom duration. In our study, 47.0% of males were current smokers, and ex-smokers were particularly prone to symptoms lasting beyond three weeks, likely due to structural and immunological alterations in the respiratory tract that impair mucosal defense and prolong inflammation. Smoking-induced airway dysfunction, characterized by bronchoconstriction and chronic inflammation, may explain the increased prevalence of wheezing observed among smokers, as prior research has linked tobacco exposure to heightened bronchial hyperresponsiveness and sustained airway remodeling (26). Additionally, the higher incidence of sputum production aligns with established evidence that smoking induces goblet cell hyperplasia and disrupts mucociliary clearance, leading to mucus accumulation and persistent cough (27). These pathophysiological changes contribute to a prolonged inflammatory state, which may, in part, explain the persistence of asthma-like symptoms and sustained elevation of inflammatory markers such as CRP even after the resolution of the acute infection. Given that smoking has lasting effects on lung function even after cessation, these findings highlight the necessity of targeted interventions for smokers presenting with post-infectious respiratory symptoms, as well as further research to delineate the mechanisms underlying prolonged inflammation in this population.

Reduced FEF25–75% is increasingly recognized as a sensitive marker for small airway dysfunction, even when traditional spirometric indices like FEV1 remain within normal limits. Studies have demonstrated that FEF25–75% more accurately reflects airway hyperresponsiveness, inflammation, and disease severity in asthma patients compared to FEV1 (13). The small airways, often termed the “silent zone” due to their asymptomatic early involvement, are a primary site for airflow limitation in asthma (12). Histopathological analyses reveal that these airways frequently exhibit chronic inflammatory infiltrates, including eosinophils, T-lymphocytes, neutrophils, and macrophages. Notably, such inflammation can persist even when FEV1 values remain stable, underscoring the importance of assessing small airway function in asthma management (14). This persistent inflammation contributes to SAD, which exacerbates bronchial hyperresponsiveness, complicates disease control, and heightens symptom frequency. Therefore, incorporating FEF25–75% measurements into routine clinical practice may enhance the detection of small airway impairment, facilitating more tailored preventative and therapeutic strategies to stop the progression of SAD into asthma (28). Although FEF25–75% is known to exhibit intra-individual variability and limited specificity, its inclusion in this study was justified by the lack of alternative small-airway metrics (e.g., impulse oscillometry, nitrogen washout, or imaging-based measures) available in retrospective datasets. When interpreted alongside normal FEV1/FVC and FEV1 values, a reduction in FEF25–75% can still serve as a surrogate indicator of distal airway dysfunction, particularly in large cohorts where consistent testing protocols minimize measurement bias. Therefore, its use here provides a pragmatic and standardized means to screen for small airway involvement in postinfectious airway hyperresponsiveness.

In elderly patients, prominent bronchovascular markings observed on chest X-rays are often associated with prolonged dyspnea, suggesting significant airway involvement. Studies have shown that aging is independently associated with increased bronchial hyperresponsiveness, even in the absence of overt pulmonary disease. This hyperresponsiveness can lead to temporary bronchial constriction, exacerbating respiratory symptoms (6). Additionally, allergic inflammation, marked by elevated IgE levels, contributes to structural airway changes and hyperreactivity (15). The coexistence of upper and lower airway diseases, sharing IgE-mediated inflammatory pathways, further complicates the clinical picture in the elderly. Moreover, superantigens from pathogens like *Staphylococcus aureus* can induce local polyclonal IgE production, perpetuating post-infectious inflammation and bronchial hyperresponsiveness (29). Therefore, in older adults presenting with persistent respiratory symptoms and radiographic evidence of bronchovascular prominence, it is crucial to consider bronchial hyperresponsiveness as a contributing factor, necessitating comprehensive evaluation and management.

Our study highlights the critical role of demographic factors such as gender, smoking status, and age, in the accurate diagnosis and management of patients with postinfectious bronchial hyperresponsiveness. Personalized treatment strategies are essential to prevent misdiagnosis and overtreatment. Tailoring interventions based on individual patient characteristics and environmental exposures can enhance therapeutic efficacy. For instance, managing contributing factors such as GERD, controlling allergic responses, and promoting smoking cessation are pivotal in alleviating symptoms and improving quality of life. Educating patients about these aspects is crucial for preventing symptom exacerbation and reducing the risk of progression to chronic respiratory conditions. The therapeutic landscape for postinfectious cough lacks well-established guidelines; however, evidence supports the efficacy of short-term inhaled corticosteroid (ICS) therapy. A randomized, double-blind study demonstrated that treatment with extra-fine hydrofluoroalkane-beclomethasone dipropionate significantly reduced cough frequency in patients with postinfectious persistent cough compared to placebo (26). Similarly, the German Respiratory Society recommends that subacute postinfectious cough due to temporary bronchial hyperreactivity be treated with inhaled corticosteroids or inhaled beta2-agonists (8). Despite these findings, the overall therapeutic gain from ICSs is modest, and a substantial placebo effect has been observed, suggesting that the benefits may be limited to specific patient subsets (27). Therefore, judicious use of ICSs is advised, reserving them for patients with significant symptoms and limiting the duration to the minimum necessary to achieve symptom relief.

Our study delineates common characteristics observed in this patient cohort; however, the etiology of postinfectious asthma-like symptoms is likely multifactorial, involving geographic, environmental, and individual susceptibility factors. Environmental and occupational exposures, including air pollution, humidity, allergens, and workplace irritants, were not systematically recorded, thus potential confounding effects on symptom persistence could not be excluded. These unmeasured variables may have contributed to the observed heterogeneity in respiratory symptoms and inflammatory markers. Randomized controlled trials or geographically matched cohort studies are needed to identify patient subgroups that would derive the most benefit from short-term ICS therapy. A notable challenge in managing this patient population is the absence of a specific ICD code for postinfectious bronchial hyperresponsiveness. The development of a precise ICD code encompassing postinfectious airway hyperresponsiveness would facilitate appropriate treatment and reduce reliance on potentially misleading diagnostic labels. Such research could inform the development of tailored treatment protocols and potentially lead to the establishment of a dedicated ICD code for postinfectious conditions, thereby promoting more accurate and effective management strategies.

Several methodological limitations warrant consideration. First, asthma exclusion in this study was based on clinical history and normal spirometry (FEV₁/FVC ≥ 0.70, normal FEV₁ and FVC), which does not fully satisfy GINA diagnostic criteria. Advanced confirmatory tests such as bronchial challenge, FeNO measurement, or serial peak-flow monitoring were not available in this retrospective dataset, introducing the possibility of misclassification, particularly in patients with cough-variant or mild asthma phenotypes. Second, the infective etiology of the preceding URI could not be determined, as pathogen-specific testing was not available in this retrospective dataset. This limitation precludes stratification by viral or bacterial cause, which may influence both symptom duration and degree of postinfectious airway inflammation. Future studies incorporating pathogen confirmation would clarify whether certain viral or bacterial infections preferentially trigger prolonged bronchial hyperresponsiveness. Third, the absence of comparator groups (healthy individuals and confirmed asthma patients) limits our ability to determine whether the observed features, such as reduced FEF₂₅–₇₅% with preserved FEV₁/FVC, are specific to postinfectious airway dysfunction or reflect broader small-airway pathology. Fourth, longitudinal follow-up data were unavailable, preventing confirmation of symptom resolution or persistence beyond the index visit; thus, the assumption of a “transient” postinfectious course remains inferential. Finally, because of these combined constraints, the present findings should be interpreted as hypothesis-generating rather than confirmatory. Future prospective studies incorporating standardized diagnostic testing (bronchial challenge, FeNO), control cohorts, and follow-up assessments at 3–12 months are required to validate these observations and to better delineate transient postinfectious airway hyperresponsiveness from evolving chronic airway disease.

### The post-infectious phenotype

Based on our analysis, the typical profile of Post-Infectious Bronchial Hyperresponsiveness (PIBHR) is characterized by a cough persisting for 3–8 weeks following an upper respiratory infection (8). Spirometric evaluation typically reveals a normal FEV1/FVC ratio, though a reduced FEF25-75 often indicates underlying small airway involvement (9). Biochemically, these patients present with normal IgE levels and eosinophil counts, while chest X-rays are generally normal, with the exception of transient bronchovascular prominence occasionally observed in elderly patients. For patients matching this specific profile, we recommend avoiding the diagnostic label of “asthma” and the initiation of long-term controller therapy, unless symptoms persist beyond eight weeks or there is demonstrable objective variability in airflow.

For the clinical decision-making, authors of the study suggest the following approach based on the current evidence:

For adults presenting after an upper respiratory infection with normal spirometry and no signs of active infection:

1. Provide reassurance and watchful waiting for subacute cough (≈3–8 weeks);
2. Consider a short, defined trial (e.g., 2–4 weeks) of inhaled bronchodilator and/or inhaled corticosteroid for bothersome symptoms;
3. Reassess at 6–8 weeks, if persistent, evaluate for asthma (including bronchial challenge or FeNO where available) and alternative diagnoses;
4. Avoid long-term controller therapy unless chronic disease is confirmed.

To mitigate missed early asthma in practice, patients managed conservatively for suspected PIBHR should be reassessed at 6–8 weeks; persistence/recurrence of symptoms should prompt objective testing where available (bronchial challenge, FeNO, or serial peak-flow variability) before initiating long-term controller therapy.

## Conclusion and future perspective

Precision in diagnosis is essential for distinguishing transient postinfectious bronchial hyperresponsiveness from chronic respiratory conditions such as asthma, ensuring that patients receive appropriate and effective care. This study underscores the need for integrating clinical assessments with objective diagnostic tools—such as spirometry for functional evaluation and radiologic imaging for detecting structural airway changes—to improve diagnostic accuracy and guide tailored treatment strategies. Such an approach minimizes the risk of misdiagnosis and overtreatment, particularly given the lack of a dedicated ICD classification for postinfectious airway dysfunction. While short-term inhaled corticosteroids have shown benefits in managing postinfectious cough, the therapeutic gain is modest, and their indiscriminate use should be avoided, necessitating further investigation into which patients truly benefit from such interventions.

Future research should focus on long-term follow-up to determine the persistence of symptoms, evaluate the risk of progression to chronic conditions, and identify biomarkers that differentiate transient inflammation from evolving airway disease. Longitudinal studies assessing the natural history of postinfectious airway hyperresponsiveness will be crucial in identifying high-risk populations and determining whether persistent symptoms lead to long-term airway remodeling. Ultimately, refining diagnostic criteria, improving treatment precision, and developing standardized clinical guidelines will facilitate a more structured and individualized approach to managing postinfectious bronchial hyperresponsiveness, improving patient outcomes while reducing unnecessary medical interventions.

## Data Availability

The original contributions presented in the study are included in the article/Supplementary material, further inquiries can be directed to the corresponding author.

## Glossary

URIs: Upper respiratory tract infections
IRB: Institutional Review Board
WBC: White blood cell count
HGB: Hemoglobin
HCT: Hematocrit
MCV: Mean corpuscular volume
IgE: Immunoglobulin E
EOS: Eosinophil count
NEUT: Neutrophil count
LYM: Lymphocyte count
CRP: C-reactive protein level
SLE: Systemic lupus erythematosus
AS: Ankylosing spondylitis
RA: Rheumatoid arthritis
GERD: Gastroesophageal reflux disease
PFTs: Pulmonary function tests
ATS: American Thoracic Society
ERS: European Respiratory Society
FEV1: Forced expiratory volume in 1 second
FVC: Forced vital capacity
FEF25-75%: Forced expiratory flow rate at 25–75% of the vital capacity
GOLD: Global Initiative for Chronic Obstructive Lung Disease
SAD: Small Airways Disease
