# Supplementary material for: Clinical and demographic characteristics of patients presenting with post-infectious bronchial hyperresponsiveness at a pulmonology clinic
Source: Front Med (Lausanne). 2026 Jan 12;12:1632712. doi: 10.3389/fmed.2025.1632712 (PMC12833338; doi:10.3389/fmed.2025.1632712)
Supplement: Supplementary file 1 [file Table_1.docx]

**Supplementary Table 1: Association between  smoking status and demographics and clinical characteristics of the study population**

| **Clinical Characteristic** | **Non-smoker (n, %)** | **Smoker (n, %)** | **Former Smoker (n, %)** | **p-value** |
| --- | --- | --- | --- | --- |
| **Age Groups** |  |  |  | **.001***** |
| 18-24 | 34 (6.5%) | 34 (6.3%) | 1 (1.2%) |  |
| 25-34 | 136 (25.9%) | 176 (32.7%) | 15 (18.1%) |  |
| 35-44 | 155 (29.5%) | 172 (31.9%) | 16 (19.3%) |  |
| 45-54 | 89 (17.0%) | 95 (17.6%) | 16 (19.3%) |  |
| 55-64 | 55 (10.5%) | 46 (8.5%) | 24 (28.9%) |  |
| 65+ | 56 (10.7%) | 16 (3.0%) | 11 (13.3%) |  |
| Total | 525 (45.8%) | 539 (47.0%) | 83 (7.2%) |  |
| **Gender** |  |  |  | **.025*** |
| - Female | 351 (66.9%) | 306 (56.8%) | 44 (53.0%) |  |
| - Male | 174 (33.1%) | 233 (43.2%) | 39 (47.0%) |  |
| **Wheezing** |  |  |  | **.001***** |
| - No | 482 (91.8%) | 451 (83.8%) | 73 (88.0%) |  |
| - Yes | 43 (8.2%) | 87 (16.2%) | 10 (12.0%) |  |
| **Sputum** |  |  |  | **.005**** |
| - No | 358 (68.2%) | 305 (56.6%) | 53 (63.9%) |  |
| - Yes | 167 (31.8%) | 234 (43.4%) | 30 (36.1%) |  |
| **Dyspnea** |  |  |  | .152 |
| - No | 339 (64.6%) | 339 (62.9%) | 58 (69.9%) |  |
| - Yes | 186 (35.4%) | 200 (37.1%) | 25 (30.1%) |  |
| **HGB Levels** |  |  |  | **.019*** |
| - Below Reference | 57 (19.3%) | 37 (11.8%) | 11 (23.9%) |  |
| - Within Reference | 230 (78.0%) | 251 (80.2%) | 33 (71.7%) |  |
| - Above Reference | 8 (2.7%) | 25 (8.0%) | 2 (4.3%) |  |
| **IgE** |  |  |  | **.042*** |
| - Within Reference | 75 (68.8%) | 54 (52.4%) | 7 (70.0%) |  |
| - Above Reference | 34 (31.2%) | 49 (47.6%) | 3 (30.0%) |  |

**p ≤ 0.05, **p ≤ 0.01, ***p ≤ 0.001*

**Supplementary Table 2: Association Between Allergy History and Demographics and Clinical Characteristics of the Study Population**

| **Clinical Characteristic** | **No Allergy (n, %)** | **Allergy (n, %)** | **p-value** |
| --- | --- | --- | --- |
| **Gender** |  |  | **.007**** |
| - Female | 252 (57.8%) | 569 (65.4%) |  |
| - Male | 184 (42.2%) | 301 (34.6%) |  |
| **Age Group** |  |  | .183 |
| - 18-24 | 28 (6.4%) | 52 (6.0%) |  |
| - 25-34 | 105 (24.1%) | 267 (30.7%) |  |
| - 35-44 | 131 (30.0%) | 251 (28.9%) |  |
| - 45-54 | 85 (19.5%) | 144 (16.6%) |  |
| - 55-64 | 48 (11.0%) | 95 (10.9%) |  |
| - 65+ | 39 (8.9%) | 61 (7.0%) |  |
| **Smoking Status** |  |  | .654 |
| - Non-smoker | 165 (43.9%) | 360 (46.7%) |  |
| - Smoker | 182 (48.4%) | 357 (46.3%) |  |
| - Former smoker | 29 (7.7%) | 54 (7.0%) |  |
| **Cough** |  |  | **.018*** |
| - Yes | 366 (28.4%) | 771 (59%) |  |
| **Wheezing(symptom)** |  |  | .701 |
| - No | 284 (65.1%) | 576 (66.2%) |  |
| - Yes | 152 (34.9%) | 294 (33.8%) |  |
| **Post-nasal Drip** |  |  | **.001***** |
| - No | 330 (75.7%) | 524 (60.2%) |  |
| - Yes | 106 (24.3%) | 346 (39.8%) |  |
| **Sputum** |  |  | .**038*** |
| - No | 299 (68.6%) | 546 (62.8%) |  |
| - Yes | 137 (31.4%) | 324 (37.2%) |  |
| **Dyspnea** |  |  | .337 |
| - No | 278 (63.8%) | 578 (66.4%) |  |
| - Yes | 158 (36.2%) | 292 (33.6%) |  |
| **Symptom Duration** |  |  | **.045*** |
| - < 3 Weeks | 206 (47.3%) | 621 (71.4%) |  |
| - ≥ 3 Weeks | 230 (52.7%) | 249 (28.6%) |  |
| **Rales** |  |  | .110 |
| - No | 256 (58.6%) | 529 (60.6%) |  |
| - Yes | 181 (41.4%) | 344 (39.4%) |  |
| **Rhonchi** |  |  | .256 |
| - No | 305 (69.8%) | 605 (69.2%) |  |
| - Yes | 132 (30.2%) | 270 (30.8%) |  |
| **Wheezing (Physical Exam Finding)** |  |  | .646 |
| - No | 284 (65.1%) | 576 (66.2%) |  |
| - Yes | 152 (34.9%) | 294 (33.8%) |  |
| **Chest X-ray** |  |  | .065 |
| - Normal | 280 (64.2%) | 524 (60.2%) |  |
| - Abnormal | 156 (35.8%) | 346 (39.8%) |  |

**p ≤ 0.05, **p ≤ 0.01*

**Supplementary Table 3: Association between bronchovascular prominence on chest X-ray images and demographics and clinical characteristics of the study population**

| **Clinical Characteristic** | **Bronchovascular Prominence Present (n)** | **Bronchovascular Prominence Present (%)** | **p-value** |
| --- | --- | --- | --- |
| **Age Group** |  |  | **.001***** |
| 18-24 | 2 | 3.6% |  |
| 25-34 | 13 | 4.9% |  |
| 35-44 | 17 | 6.6% |  |
| 45-54 | 28 | 18.5% |  |
| 55-64 | 10 | 13.9% |  |
| 65+ | 11 | 21.6% |  |
| **Gender** |  |  | .065 |
| Female | 41 | 8.0% |  |
| Male | 40 | 11.7% |  |
| **Smoking Status** |  |  | .697 |
| Non-smoker | 34 | 10.5% |  |
| Smoker | 34 | 8.7% |  |
| Former Smoker | 6 | 10.3% |  |
| **Symptoms Duration** |  |  | .842 |
| <3 Weeks | 58 | 9.3% |  |
| ≥3 Weeks | 23 | 9.8% |  |
| **Wheezing (clinical finding)** | 7 | 7.1% | .385 |
| **Wheezing (symptom)** | 26 | 8.6% | .544 |
| **Cough** | 67 | 9.0% | .210 |
| **Dyspnea** | 40 | 13.7% | **.002**** |
| **Sputum** | 34 | 10.6% | .382 |
| **IgE** |  |  | **.035*** |
| - Within Reference | 4 | 3.6% |  |
| - Above Reference | 9 | 11.4% |  |
| **History of Diabetes Mellitus** | 8 | 22.2% | **.008**** |

**p ≤ 0.05, **p ≤ 0.01*

**Supplementary Table 4: Association between blood eosinophil levels and demographics and clinical characteristics of the study population**

| **Clinical Characteristic** | **EOS Levels**  - Below Reference | **EOS Levels**    - Within Reference | **EOS Levels**   - Above Reference | **p-value** |
| --- | --- | --- | --- | --- |
| **Wheezing Symptom (n,%)** | 12(4,8%) | 186 (73,8%) | 54 (21,4%) | **.003**** |
| **IgE** |  |  |  | .445 |
| - Within Reference | 7 (4.8%) | 115 (78.2%) | 25 (17.0%) |  |
| - Above Reference | 7 7.7%) | 65 (71.4%) | 19 (20.9%) |  |

**p ≤ 0.05, **p ≤ 0.01*
